# Clinical effect of short-term spinal cord stimulation in the treatment of patients with primary brainstem hemorrhage-induced disorders of consciousness

**DOI:** 10.3389/fneur.2023.1124871

**Published:** 2023-03-17

**Authors:** Weilong Huang, Qiang Chen, Lin Liu, Jianhong Tang, Hua Zhou, Zhiji Tang, Qing Jiang, Tao Li, Jianwu Liu, Dong Wang

**Affiliations:** ^1^Department of Neurosurgery, Ganzhou People's Hospital, Ganzhou, China; ^2^Key Laboratory of Prevention and Treatment of Cardiovascular and Cerebrovascular Diseases of Ministry of Education, Gannan Medical University, Ganzhou, China; ^3^Laboratory Animal Engineering Research Center of Ganzhou, Gannan Medical University, Ganzhou, China

**Keywords:** short-term spinal cord stimulation, primary brainstem hemorrhage, disorder of consciousness, minimally conscious state, neuromodulation

## Abstract

**Objective:**

Recently, short-term spinal cord stimulation (st-SCS) has been used in neurorehabilitation and consciousness recovery. However, little is known about its effects on primary brainstem hemorrhage (PBSH)-induced disorders of consciousness (DOC). In this study, we examined the therapeutic effects of st-SCS in patients with PBSH-induced DOC.

**Methods:**

Fourteen patients received a 2-week st-SCS therapy. Each patient's state of consciousness was evaluated using the Coma Recovery Scale-Revised (CRS-R). CRS-R evaluation scores were recorded at the baseline (before SCS implantation) and 14 days later.

**Results:**

Over 70% (10/14) of the patients (CRS-R score increased to ≥2 points) responded to the SCS stimulation after 14 days of st-SCS treatment. All items included in the CRS-R exhibited a significant increase post-treatment compared with pretreatment. After 2 weeks of st-SCS treatment, seven patients showed diagnostic improvement, resulting in a 50% (7/14) overall effective rate. Approximately 75% (3/4) of patients with minimally conscious state plus (MCS+) improved to emergence from MCS (eMCS), and 50% (1/2) of patients with vegetative state or unresponsive wakefulness syndrome (VS/UWS) improved to MCS+.

**Conclusion:**

In PBSH-induced DOC, st-SCS is a safe and effective treatment. The clinical behavior of the patients improved significantly following the st-SCS intervention, and their CRS-R scores markedly increased. This was most effective for MCS+.

## 1. Introduction

Primary brainstem hemorrhage (PBSH) is a hemorrhagic stroke subtype that occurs in the pons in the vast majority of cases and accounts for ~5%−10% of intracerebral hemorrhage cases (1–3). This disease is characterized by an abrupt onset of symptoms, rapid neurological decline, poor prognosis, and high mortality (30%−90%) (4–6). Currently, the main therapeutic options for PBSH are conservative treatments, but surgical interventions have become increasingly attractive as treatment options (7, 8). Surgical removal of hematomas can achieve hemostasis, relieve brainstem pressure, and prevent secondary damage (9–11). However, abnormal rupture of blood vessels in brainstem-induced brain injuries can result in severe disorders of consciousness (DOC), often with a serious impact on postoperative recovery (12). Thus, the development of effective strategies targeting PBSH-induced DOC would be beneficial in clinical treatment.

Interest has increased concerning DOC, which is caused by severe brain injuries that cause loss or partial loss of consciousness (13, 14). The term disorders of consciousness summarize the vegetative state or unresponsive wakefulness syndrome (VS/UWS), minimally conscious state (MCS), and then emergence from the minimally conscious state (eMCS) (15, 16). VS/UWS is a severe DOC, defined as a state of unresponsiveness in which the patient shows spontaneous eye opening without any behavioral evidence of awareness of either the self or environment (17). MCS is defined as a state of severely impaired consciousness with minimal behavioral evidence of self or environmental awareness, manifested as the presence of non-reflexive behaviors (visual pursuit, appropriate motor response to a painful stimulus) or even intermittent command following cortical integration (16, 18). Thus, patients in MCS usually show a stronger level of awareness than those in VS/UWS, and the Coma Recovery Scale-Revised (CRS-R) has been recommended as the assessment scale (19, 20). Furthermore, with increasing research on MCS, it has been possible to divide MCS into minimally conscious state minus (MCS–) and minimally conscious state plus (MCS+) (21). The difference between the two is that the former displays low-level consciousness responses, whereas the latter demonstrates language-related cognitive abilities (22). Patients with MCS+ show high-level behavioral responses (i.e., command following, intelligible verbalizations, or non-functional communication), and patients with MCS– have low-level behavioral responses (i.e., visual pursuit, localization of noxious stimulation, or contingent behavior such as appropriate smiling or crying to emotional stimuli) (23). In addition, patients are classified as emerging from MCS (eMCS) when the patient can communicate functionally or show proper functional objects (24, 25).

The treatment of DOC still lacks a curative strategy. Several new non-invasive neuromodulation treatments have been developed in recent years, including transcranial direct current stimulation (tDCS) and repetitive transcranial magnetic stimulation (rTMS) (26–28). According to recent studies, loss of consciousness after severe brain injury is closely related to the disruption of neural circuits (such as cortico-thalamic and cortico-cortical connections) (29). According to its principles, non-invasive neuromodulation therapy does not directly modulate the neural circuit, particularly the cortico-thalamic connection. Thus, spinal cord stimulation (SCS) has become an essential and valid surgical treatment for DOC because of its relative ease of operation, safety, wide range of indications, effectiveness, and direct modulation of neural circuits (30). However, there are many difficulties in applying SCS to the clinical treatment of DOC, such as significant injuries caused by invasive operations and potential implant rejection. Therefore, SCS is usually used to treat patients with DOC with a disease duration of more than 3 months to avoid spontaneous high-speed recovery of consciousness (31). A previous study found that early rehabilitation was crucial for patients with DOC (32). Therefore, short-term spinal cord stimulation (st-SCS) has been developed. Another study already applied this method for the recovery from DOC (33, 34), but it was unclear whether it affected PBSH-induced DOC.

In this study, we hypothesized that st-SCS would improve the recovery of consciousness in patients with PBSH. We studied 14 patients with PBSH-induced DOC, diagnosed using the CRS-R test, and treated with st-SCS.

## 2. Materials and methods

### 2.1. Participants

Fourteen patients (nine men and five women; mean age, 55.79 ± 8.29 years) with MCS or VS/UWS who underwent st-SCS treatment in our hospital from November 2021 to July 2022 were enrolled. Ten of the 14 patients underwent minimally invasive stereotactic puncture therapy (MISPT) before st-SCS treatment. The average time since injury was 1.27 ± 0.31 months and ranged from 1 to 1.7 months. Detailed clinical information for each patient is presented in Table 1. We recruited patients who met the following inclusion criteria: (1) age ≥18 years with the onset of PBSH; (2) at least one neurological examination consistent with DOC defined by the CRS-R test; and (3) written informed consent obtained from legal surrogates. The exclusion criteria were as follows: (1) other intracerebral hemorrhage conditions; (2) age < 18 years; (3) disagreement of relatives or their legal representative with MCS treatment; and (4) poor condition (other vital organ dysfunction or severe infection) and surgical inoperability. The Ethics Committee of Ganzhou People's Hospital approved the study protocol.

**Table 1:** Clinical patient information.

**No**.	**Gender**	**Age (years)**	**Cause**	**MISPT (yes/no)**	**Post-injury (months)**	**Diagnosis**
1	Male	48	PBSH	No	1	MCS–
2	Male	42	PBSH	No	1.5	MCS+
3	Female	66	PBSH	Yes	1.7	MCS+
4	Male	48	PBSH	Yes	1	MCS–
5	Male	53	PBSH	No	1.3	MCS–
6	Female	51	PBSH	No	1.7	MCS–
7	Male	64	PBSH	Yes	1	MCS–
8	Male	68	PBSH	Yes	1.3	MCS–
9	Female	69	PBSH	Yes	1	VS/UWS
10	Female	58	PBSH	Yes	1.6	MCS–
11	Male	49	PBSH	Yes	1	VS/UWS
12	Male	54	PBSH	Yes	1	MCS–
13	Female	56	PBSH	Yes	1.7	MCS+
14	Male	55	PBSH	Yes	1	MCS+

MISPT, minimally invasive stereotactic puncture therapy; PBSH, primary brainstem hemorrhage; VS/UWS, vegetative state or unresponsive wakefulness syndrome; MCS–, minimally conscious state minus; MCS+, minimally conscious state plus; eMCS, emerged from MCS.

### 2.2. SCS implantation

**Figure 1:**
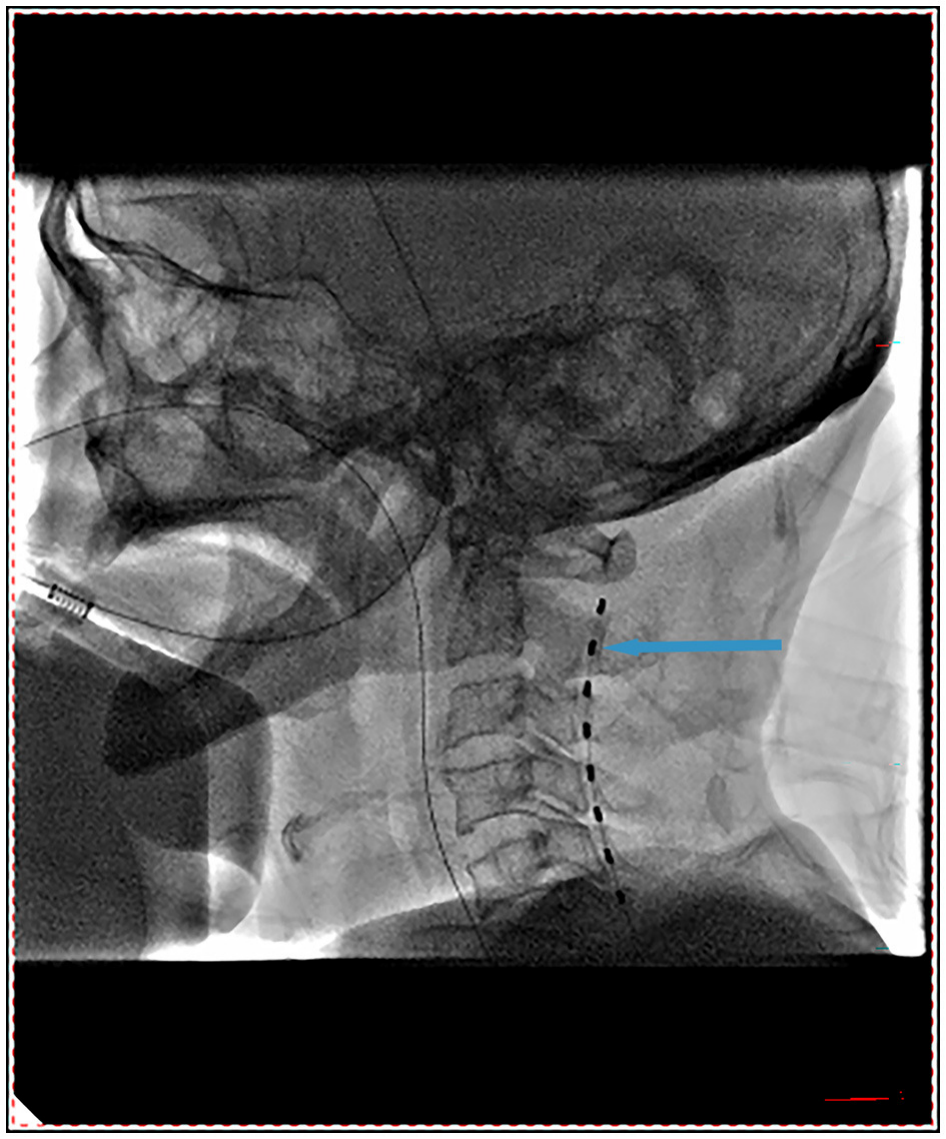
Electrode position during the operation.

Before SCS implantation, all patients underwent the following preoperative routine examinations: medical history, imaging examinations, and routine laboratory tests. Following the screening, all eligible patients were included in the study to receive SCS system (Medtronic Inc., Minneapolis, USA) implantation, as previously described (33, 34). Following general anesthesia, the patients were placed in a prone position and their necks were flexed forward. An 8-contact stimulation electrode (3777; Medtronic, Minneapolis, MN, USA) was inserted into the epidural spaces of T7 and T8. Next, the test stimulation lead was placed under X-ray fluoroscopy, and the electrode was flattened on the upper edge of the cervical-2 vertebral body (Figure 1). Finally, the electrode was properly fixed, the multi-lead trialing cable was connected, an external neurostimulator was connected to the assembly, and test stimulation was performed intraoperatively to maintain the best state of the machine.

### 2.3. Adjustment of st-SCS parameters

After the st-SCS operation, the electrical stimulation of the spinal cord lasted for 14 days, and the electrode was removed. From 8 a.m. to 8 p.m., 5-min on/15-min off cycles were performed. The machine was turned on with the following parameters: voltage, 2.0 V; frequency, 70 Hz; and pulse width, 210 μs.

### 2.4. Behavioral assessment

The Chinese version of the CRS-R scale was used to assess the patient's state during the entire st-SCS treatment protocol (35, 36). The CRS-R consists of six subscales with total scores ranging from 0 to 23. The scoring standards for the CRS-R scale are presented in Table 2.

**Table 2:** Description of items included in the CRS-R.

**Item**	**CRS-R**	**Diagnosis**
Auditory	4-Consistent movement to command	MCS+
	3-Reproduction movement to command	MCS+
	2-Sound localization	
	1–1 Auditory startling	
	0-None	
Visual	5-Object recognition	MCS+
	4-Object localization (reaching)	MCS–
	3-Visual pursuit	MCS–
	2-Fixation (>2 s)	
	1-Visual startle (startle reaction)	
	0-None	
Motor	6-Functional object use	eMCS
	5-Automatic motor response	MCS–
	4-Object manipulation	MCS–
	3-Flexion to noxious stimulation	MCS–
	2-Flexion withdraw	
	1-Abnormal posturing	
	0-None	
Oromotor	3-Intelligible verbalization	MCS+
	2-Vacalization	
	1-Oral reflexive movement	
	0-None	
Communication	2-Functional (accurate)	eMCS
	1-Non-functional	MCS+
	0-None	
Arousal level	3-Attention	
	2-Eye opening	
	1-Eye opening with stimulation	
	0-None	

CRS-R, Coma Recovery Scale-Revised; MCS+, minimally conscious state plus; MCS–, minimally conscious state minus; eMCS, emerged from MCS.

The CRS-R assessments were administered by clinicians who were not responsible for the st-SCS treatment. A minimum of six CRS-R assessments were recorded before the operation and 14 days after st-SCS therapy (35). The CRS-R scores for each patient in this study were based on their best responses to repeated CRS-R assessments (37). The effective clinical outcome of st-SCS was that patients showed a CRS-R score improvement. Patients with positive st-SCS responses exhibited an increase of ≥2 points in the CRS-R. In irresponsive patients, the total CRS-R scores remained unchanged or increased by < 2 (38). Safety was primarily assessed by analyzing treatment-emergent adverse events (TEAEs).

### 2.5. Statistical analysis

Statistical results were demonstrated using an online scientific analysis platform, SPSSAU (version 20.0; Beijing, China, https://www.spssau.com). Categorical data and univariate analysis results were analyzed using Fisher's exact test, Mann–Whitney *U*-test, and Wilcoxon matched-pairs signed-rank test. A significant difference was defined as a *p*-value of < 0.05. The statistical parameters for each analysis can be found in the relevant figure legends.

## 3. Results

### 3.1. Feasibility and safety

Fourteen patients (nine men and five women; mean age, 55.79 ± 8.29 years) with DOC who underwent st-SCS were enrolled in this study. The average time since injury was 1.27 ± 0.31 months and ranged from 1 to 1.7 months. All cases of consciousness in this study were due to PBSH (Table 1). Of all 14 patients, 10 were treated with minimally invasive stereotactic puncture therapy (MISPT) before SCS implantation. Notably, we did not record any severe adverse events (such as seizures or intracranial infections) associated with st-SCS implantation or programming.

### 3.2. Clinical diagnostic changes after st-SCS treatment

**Figure 2:**
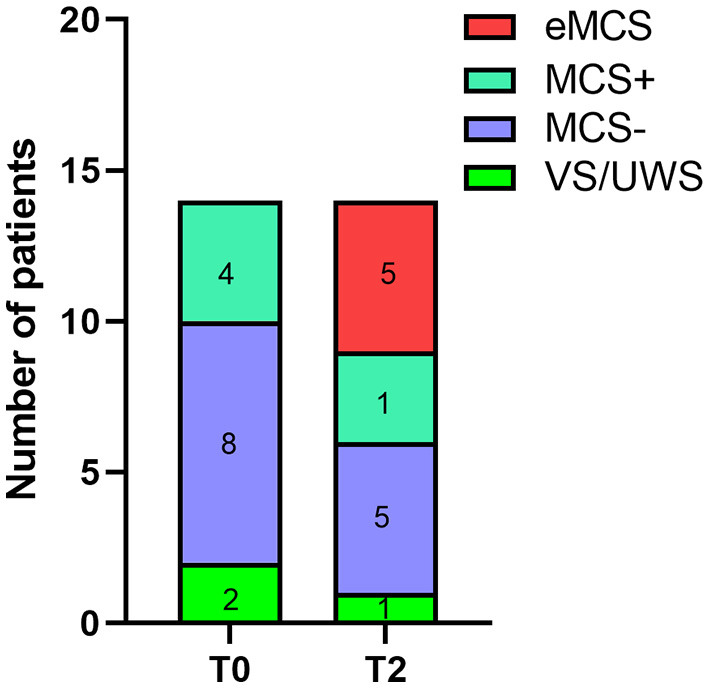
Changes in clinical diagnosis before and after treatment. T0, before the treatment; T2, 2 weeks of follow-up.

After 2 weeks of st-SCS treatment, seven patients had improved diagnostic results, with an overall effectiveness rate of 50% (7/14) (Table 3). An effective rate of 50% (6/12) was found in the patients with MCS, and a 50% (1/2) effective rate was also found in the patients with VS/UWS. After analyzing the clinical sample information, we found that 75% (3/4) of patients with MCS+ improved to eMCS, 50% (1/2) of those with VS/UWS improved to MCS+, 25% (2/8) of those with MCS– improved to eMCS, and only 12% (1/8) of those with MCS– improved to MCS+ (Table 3 and Figure 2).

**Table 3:** Clinical data of patients with disorders of consciousness treated by short-term spinal cord stimulation.

**No**.	**CRS-R (T0)**	**CRS-R (T2)**	**Changes of diagnosis**
1	8 (0–3–2–1–0–2)	20 (4–4–5–1–2–3)	MCS– improved to eMCS
2	14 (3–3–3–1–1–3)	23 (4–5–6–3–2–3)	MCS+ improved to eMCS
3	15 (3–3–4–1–1–3)	19 (4–5–6–1–1–3)	MCS+ improved to eMCS
4	4 (0–3–1–0–0–0)	6 (0–3–1–1–0–1)	Remained MCS–
5	6 (1–3–0–0–0–2)	8 (1–3–2–0–0–2)	Remained MCS–
6	8 (1–3–2–1–0–1)	10 (2–3–2–1–0–2)	Remained MCS–
7	8 (1–3–2–0–0–2)	23 (4–5–6–3–2–3)	MCS– improved to eMCS
8	8 (1–3–2–1–0–1)	8 (1–3–2–1–0–1)	Remained MCS–
9	5 (1–2–0–1–0–1)	11 (3–3–2–1–0–2)	VS/UWS improved to MCS+
10	8 (1–3–2–0–0–2)	14 (3–3–3–1–1–3)	MCS– improved to MCS+
11	5 (1–0–2–0–0–2)	5 (1–0–2–0–0–2)	Remained VS/UWS
12	8 (1–3–2–1–0–1)	8 (1–3–2–1–0–1)	Remained MCS–
13	19 (4–5–5–1–1–3)	21 (4–5–5–2–2–3)	MCS+ improved to eMCS
14	17 (3–3–5–1–1–3)	17 (3–3–5–1–1–3)	Remained MCS+

T0, time before SCS surgery; T2, 2 weeks after SCS surgery; VS/UWS, vegetative state or unresponsive wakefulness syndrome; MCS–, minimally conscious state minus; MCS+, minimally conscious state plus; eMCS, emerged from MCS; CRS-R, Coma Recovery Scale-Revised.

CRS-R includes six subscales addressing auditory, visual, motor, oromotor, communication, and arousal functions, which are summed to yield a total score ranging from 0 to 23.

### 3.3. CRS-R score changes after st-SCS therapy

**Figure 3:**
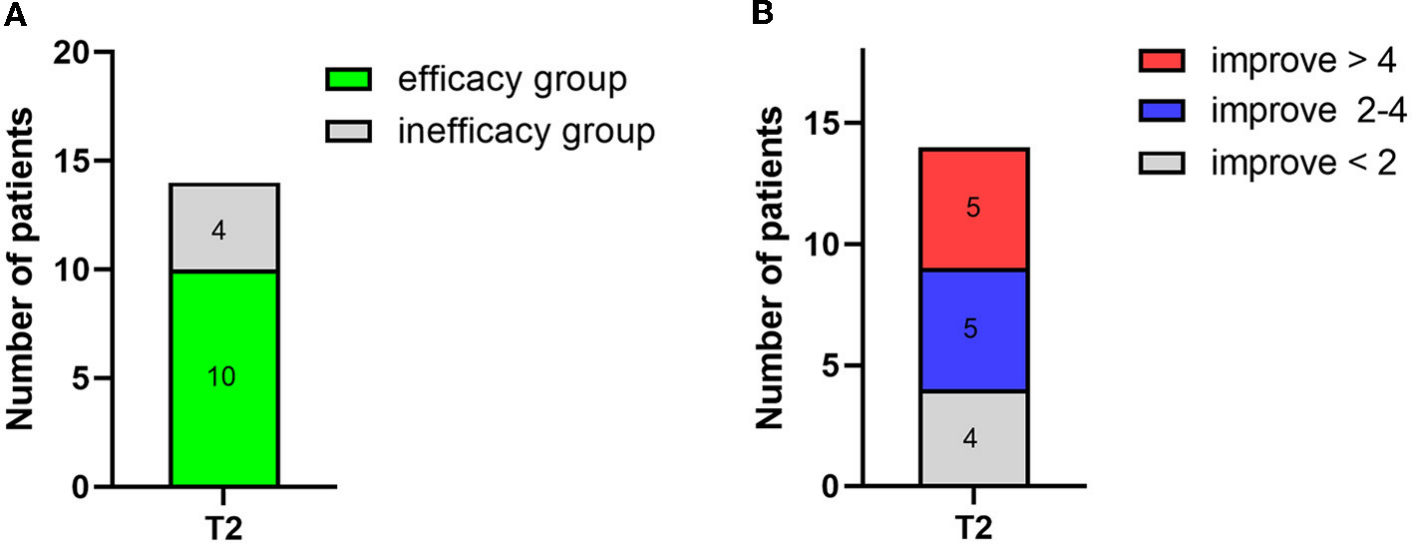
Number of patient changes in CRS-R score after 2 weeks of treatment (T2). **(A)** The number of patient changes for the efficacy group (CRS-R score increased by ≥2) and the inefficacy group (CRS-R score unchanged or increased by < 2). **(B)** Detailed number of patients and the corresponding change in CRS-R score.

Short-term spinal cord stimulation (st-SCS) treatment not only improved the clinical diagnosis of patients but also significantly improved their CRS-R scores. After 14 days of electrical stimulation, over 70% (10/14) of the patients were classified into the efficacy group (CRS-R score increased by ≥2 points), and below 30% (4/14) were classified into the inefficacy group (CRS-R score unchanged or increased by < 2 points; Figure 3A). In particular, 36% (5/14) of the patients showed an over 4-point increase, 36% (5/14) showed an increase between 2 and 4 points, and 28% (4/14) showed an increase of < 2 points (Figure 3B).

Statistical analysis of the obtained results was then performed. The statistical results showed that patients had a marked increase in their CRS-R scores after 2 weeks of st-SCS therapy (*p* = 0.005). More excitingly, all six subscales included in the CRS-R scores exhibited a significant post-treatment increase when compared with the pretreatment values (Table 4).

**Table 4:** Statistical analysis (*p*-value) of behavioral assessment by the CRS-R test.

	**T2 vs. T0**
Total CRS-R score	0.005^**^
Auditory function	0.017^*^
Visual function	0.038^*^
Motor function	0.017^*^
Oromotor	0.039^*^
Communication	0.038^*^
Arousal	0.014^*^

CRS-R, Coma Recovery Scale-Revised; T0, time before spinal cord stimulation surgery; T2, 2 weeks after spinal cord stimulation surgery.

Wilcoxon matched-pairs signed-rank test was used for all statistical analyses shown in this table.

^*^p < 0.05.

^**^p < 0.01.

In addition, clinical data from the effective and ineffective treatment groups were collected and analyzed. We assessed factors such as age, sex, and previous history of hypertension or MISPT for similarities and differences among the groups. As shown in Table 5, there were no significant differences between the two groups. Similarly, further subdivision of the MCS diagnostic revealed no significant difference between the effective and ineffective groups for the three diagnostic subgroups (VS, MCS–, and MCS+).

**Table 5:** Clinical variable comparisons between improvement and unimprovement.

**Variables**	**Improvement (*n* = 10)**	**Unimprovement (*n* = 4)**	**Statistic value**	***p*-value**
**Sex**				
Male	5	4	NA^a^	0.221
Female	5	0		
**Age (years)**				
40–60	7	3	12.780^a^	0.560
>60	3	1		
**Hypertension**				
Yes	8	3	NA^a^	1.000
No	2	1		
**MISPT**				
Yes	6	4	NA^a^	0.251
No	4	0		
Post-injure [*M* (P25, P75), days]	39.9 (30, 50)	42 (30, 38)	10.500^b^	0.149
CRS-R onset [mean (min, max)]	9.5 (4, 19)	9.5 (5, 17)	19.500^b^	0.947
**Diagnosis**				
VS/UWS	1	1	0.977^a^	1.000
MCS–	6	2		
MCS+	3	1		

MISPT, minimally invasive stereotactic puncture therapy; VS/UWS, vegetative state or unresponsive wakefulness syndrome; MCS–, minimally conscious state minus; MCS+, minimally conscious state plus.

^a^Fisher exact test.

^b^Mann–Whitney U-test.

^*^p < 0.05.

## 4. Discussion

Short-term spinal cord stimulation (St-SCS) was first used for pain relief and has become an indispensable treatment means for patients with early-stage pain (39–41). In recent years, with more extensive st-SCS investigations, it has been used in the recovery of consciousness. Our study demonstrated the safety and feasibility of st-SCS in treating PBSH-induced DOC, and it was the most effective treatment for patients with MCS+. After st-SCS treatment, over 70% of the patients showed improvement in the CRS-R score, and each item included in the CRS-R test exhibited a significant increase. Approximately 50% (7/14) of the patients showed improved neurological behavior. These results are promising for future applications of st-SCS in PBSH-induced DOC.

To the best of our knowledge, this is the first case in which st-SCS was used to treat PBSH-induced DOC. Therefore, st-SCS stimulation strategies were drawn from others reported for DOC. According to previous reports, the CRS-R score significantly increased after 2 weeks of DOC treatment at 70 Hz (33, 34); we selected this frequency for this study. According to previous studies, neuronal fatigue or damage was reduced if the stimulation time was shorter than the off-stimulation time (31). Therefore, the stimulation cycle was chosen as 5-min ON/15-min OFF. Finally, the treatment period started at 8 a.m. and ended at 8 p.m. for a total of 2 weeks to meet the patients' sleep demands. To further improve the outcome of st-SCS, future studies should consider other treatment protocols, including selected 5 Hz or prolonged treatment periods. Furthermore, non-invasive neuromodulation techniques combined with st-SCS are promising therapies for the future because they activate many brain regions simultaneously.

Furthermore, clinical data such as age, sex, and history of the disease are important for clinical treatment (42). There was no significant difference between the efficacy and inefficacy groups in terms of age, sex, hypertension, or MISPT history in our study; this result is similar to that reported in the literature (33). In addition, a subdivision of the MCS diagnosis did not reveal any significant differences between the two groups, contrary to previous research. This could be because PBSH-induced DOC may have other unclear mechanisms; moreover, the limit of sample size leading to statistical validity was not sufficient.

Finally, there were many limitations to our study, and future study is warranted. First, we used the CRS-R to diagnose DOC; however, there was also a need for neuropsychological measurements in these patients. Future studies should utilize neuroimaging and neurophysiological assessment techniques that provide objective feedback on patients' clinical performance. Second, the sample size of this study was small. The small sample size limited us from analyzing the factors that affect the therapeutic efficacy of st-SCS. Then, 3 months of follow-up were not available for some patients, limiting further statistical analysis of follow-up information. Finally, further studies are required to fully explore the mechanisms underlying st-SCS therapy.

## 5. Conclusion

In this study, we provided preliminary data suggesting that st-SCS is a safe and effective clinical therapy to facilitate the recovery of consciousness in patients with PBSH. As measured by the CRS-R score, st-SCS intervention significantly improved patients' clinical manifestations. It is worth noting that st-SCS seemed to be more applicable to patients with MCS+. Between the effective and ineffective groups, age, sex, duration of illness, and history of hypertension or MISPT had no significant effect. Further studies are required to explore whether these factors affect st-SCS therapy. The results of this study provide a new perspective on the treatment of PBSH-induced DOC with st-SCS and a reference for treating other cerebrovascular diseases.

## Data availability statement

The original contributions presented in the study are included in the article/supplementary material, further inquiries can be directed to the corresponding author.

## Ethics statement

The studies involving human participants were reviewed and approved by the Ethics Committee of Ganzhou People's Hospital. The patients/participants provided their written informed consent to participate in this study. Written informed consent was obtained from the individual(s) for the publication of any potentially identifiable images or data included in this article.

## Author contributions

DW: conceptualization and supervision. HZ and ZT: methodology. LL and JT: data curation. WH and QC: formal analysis, investigation, and writing original draft preparation. DW and JL: writing, review, and editing. DW and TL: funding acquisition. QJ: resources. All authors contributed to the writing of the article and approved the final version.
